# Environmental versus Anthropogenic Effects on Population Adaptive Divergence in the Freshwater Snail *Lymnaea stagnalis*

**DOI:** 10.1371/journal.pone.0106670

**Published:** 2014-09-10

**Authors:** Anthony Bouétard, Jessica Côte, Anne-Laure Besnard, Marc Collinet, Marie-Agnès Coutellec

**Affiliations:** INRA, UMR 0985 ESE, Ecology and Ecosystem Health, INRA – Agrocampus Ouest, CS84215, Rennes Cedex, France; Texas Tech University, United States of America

## Abstract

Repeated pesticide contaminations of lentic freshwater systems located within agricultural landscapes may affect population evolution in non-target organisms, especially in species with a fully aquatic life cycle and low dispersal ability. The issue of evolutionary impact of pollutants is therefore conceptually important for ecotoxicologists. The impact of historical exposure to pesticides on genetic divergence was investigated in the freshwater gastropod *Lymnaea stagnalis*, using a set of 14 populations from contrasted environments in terms of pesticide and other anthropogenic pressures. The hypothesis of population adaptive divergence was tested on 11 life-history traits, using *Q*_ST_ -*F*_ST_ comparisons. Despite strong neutral differentiation (mean *F*_ST_ = 0.291), five adult traits or parameters were found to be under divergent selection. Conversely, two early expressed traits showed a pattern consistent with uniform selection or trait canalization, and four adult traits appeared to evolve neutrally. Divergent selection patterns were mostly consistent with a habitat effect, opposing pond to ditch and channel populations. Comparatively, pesticide and other human pressures had little correspondence with evolutionary patterns, despite hatching rate impairment associated with global anthropogenic pressure. Globally, analyses revealed high genetic variation both at neutral markers and fitness-related traits in a species used as model in ecotoxicology, providing empirical support for the need to account for genetic and evolutionary components of population response in ecological risk assessment.

## Introduction

Understanding the causes of genetic divergence within species is a central goal in evolutionary biology. Population evolution is also a question of importance in environmental sciences and conservation biology, and, as a consequence, evolutionary principles are increasingly taken into account in the management of biodiversity and ecosystems [1]–[3]. Indeed, evolutionary change can be rapid, especially in the current environmental context. Besides evolutionary textbook cases of insecticide resistance [4], environmental stress may trigger fast evolutionary responses [5], and rapid adaptive responses have been documented in ecological situations involving anthropogenic disturbance [6], [7]. Furthermore, it is noteworthy that the rate of phenotypic change can itself increase in human-disturbed and toxic environments [8], [9]. In spite of this, the potential evolutionary impact of chemicals and toxic substances released by human activities into the environment is still not considered in current procedures of ecological risk assessment. However, strong arguments for mainstreaming such impacts have been formulated for a long time [10]–[14]. Beyond mutagenic compounds affecting the germline, pollutants have proved to be potential sources of evolutionary impact, as supported empirically by an ever growing number of studies [15]–[23].

Agriculture is probably one of the main causes of human-induced microevolution by selection, as a direct consequence of genetic resource management, e.g., domestication, selection programs on productive species, or side effects of intensive agricultural practices [24]–[26]. Furthermore, in the case of pesticides, as substances rarely reach their target species exclusively, non-intentional evolutionary effects are to be expected. Arguably, a substantial loss of biodiversity has resulted from the intensification of arable agriculture over the last five decades [27]. Agriculture, combined with industrial and domestic activities, uses more than one-third of the Earth's accessible renewable freshwater (i.e., approximately 4,430 km^3^/year in 2006) [28] and often leads to water contamination. About 140 million tons of fertilizers and several million tons of pesticides are applied each year [29]. Natural populations are thus exposed to a diversity of pressures that vary both spatially and temporally, and a succession of wildlife mortality events has marked the evolution of industry and agrichemical practices since the early 20^th^ century [30].

With respect to human-induced stress, some conditions are expected to increase the risk of genetic change in natural populations. For instance, freshwater lentic habitats located within agricultural landscapes are repeatedly contaminated by complex pesticide mixtures, through various modes of transfer from the treated parcels (including aerial drift, run-off, and drainage; see [31]). Although a given pesticide or its metabolites may be only transiently present in water, the succession and combination of different molecules are expected to lead to high environmental variation and recurrent stress. At the population level, if demographic stochasticity is triggered by stressful episodes, random genetic drift and inbreeding will increase, and impede local adaptive processes [32]. Indeed, selection efficiency is positively correlated to effective population size [33], and population response to selection is weaker under high inbreeding, due to linkage disequilibrium and selection interference among loci [34]–[36]. Also, random drift load will accumulate locally and increase the risk of population extinction [33],[37]. Furthermore, population adaptive potential is also predicted to decrease in stressful and changing environments, especially due to genetic erosion [38]. These processes will be exacerbated under limited gene flow, which may result from low dispersal abilities (fully aquatic organisms, *e.g.*, molluscs, crustaceans) or opportunities (weak connectivity among occupied sites, *e.g.*, ponds, temporary ditches) [39]. Also, population tolerance to environmental stress and change will decrease as inbreeding increases, given that inbreeding depression generally worsens under stress [40]. Interestingly, environmental heterogeneity in selection also increases inbreeding depression [41].

Alternatively, faced with stress, populations may adapt in two ways. First, they may adjust through phenotypic plasticity, which should in principle have no evolutionary consequences, although plasticity may itself be under selection [42],[43]. Second, genetic adaptation may occur, provided that adapted alleles are present and selection intensity is strong enough to overcome stochastic interference [44].

To sum up, human-induced environmental stress and heterogeneity may alter population evolutionary trajectory in varied and complex ways, the study of which is anything but an easy task. Processes described above will also have consequences on population genetic divergence: both random genetic drift and local adaptation will cause population divergence, whereas environmental stress and heterogeneity may either act in the same way (random drift or varying selection regimes) or on the contrary, trigger similar adaptive responses among populations (uniform selection acting on general stress responsive pathways or traits).

One method commonly used to decipher population genetic divergence in terms of selective and neutral processes, is the *Q*_ST_-*F*_ST_ approach [45],[46]. The general principle is to partition the total genetic variance at neutral markers and in quantitative traits into within- and between-population components, using Wright's *F*_ST_ index, and its quantitative analogue *Q*_ST_, respectively. Under a purely additive determinism, if quantitative traits evolve neutrally, they should lead to similar levels of differentiation as those inferred from neutral markers (*Q*_ST_  =  *F*_ST_). Basically, *Q*_ST_ and *F*_ST_ are compared to test the null hypothesis of neutrality. Differences between the two indices indicate selection on the traits, with two possibilities: homogenizing (uniform stabilizing) selection towards a unique fitness optimum is revealed by *F*_ST_>*Q*_ST_, whereas divergent selection (occurrence of different fitness optima among populations) is reflected by the reverse relationship. This principle has been widely used in the last decade to address population adaptive divergence (see [46] and references therein). It has notably revealed population adaptive divergence as a function of habitat [47], environmental acidification [48], or soil contamination by zinc [49].

In the present study, we applied the *Q*_ST_-*F*_ST_ method to test the hypothesis that anthropogenic pressure may act as a selective force and lead to population adaptive divergence, using a set of 14 natural populations of the freshwater snail *Lymnaea stagnalis* (Mollusca, Gastropoda, Panpulmonata, Hygrophila; previously classified in the Sub-Order Basommatophora). Due to its habitat characteristics (lentic systems, often close to agricultural zones) and its limited dispersal ability, this species is a good model to test non-intentional evolutionary effects of pesticides. Moreover, *L. stagnalis* belongs to an ecologically important group, which plays a major role in the transfer of energy across food webs, and can represent up to 20–60% of the total biomass of macro-invertebrates in some freshwater ecosystems [50]. Pesticides and other identified anthropogenic pressures were described qualitatively and considered as contributing to a global and potentially toxic pressure on non-target organisms. In this context, directional selection associated with a particular pesticide was considered irrelevant, and thus not specifically focused. Conversely, we hypothesized that global chronic exposure to various cocktails of pesticides may induce selective response to temporary but recurrent stress. The study aimed at addressing the following questions: (i) is agricultural (pesticide) pressure able to trigger adaptive processes in non-target organisms? (ii) what is the relative strength of such a pressure, compared to other environmental components, including natural and other anthropogenic factors? Indeed, as natural populations in a given level of pesticide pressure are not eco-evolutionary replicates of each other, any other relevant environmental factor should be also considered. Under this rationale, we characterized environments in terms of “pesticide pressure”, “other anthropogenic pressures” (roads, urbanized zones), “global pressure” (combination of agricultural and non agricultural anthropogenic pressures), and “habitat” (pond, ditch, channel, as physical features expected to affect population size and isolation). Population divergence patterns were investigated for a set of life-history traits and compared to neutral genetic differentiation.

## Materials and Methods

### Population sampling and characterization

**Figure 1:**
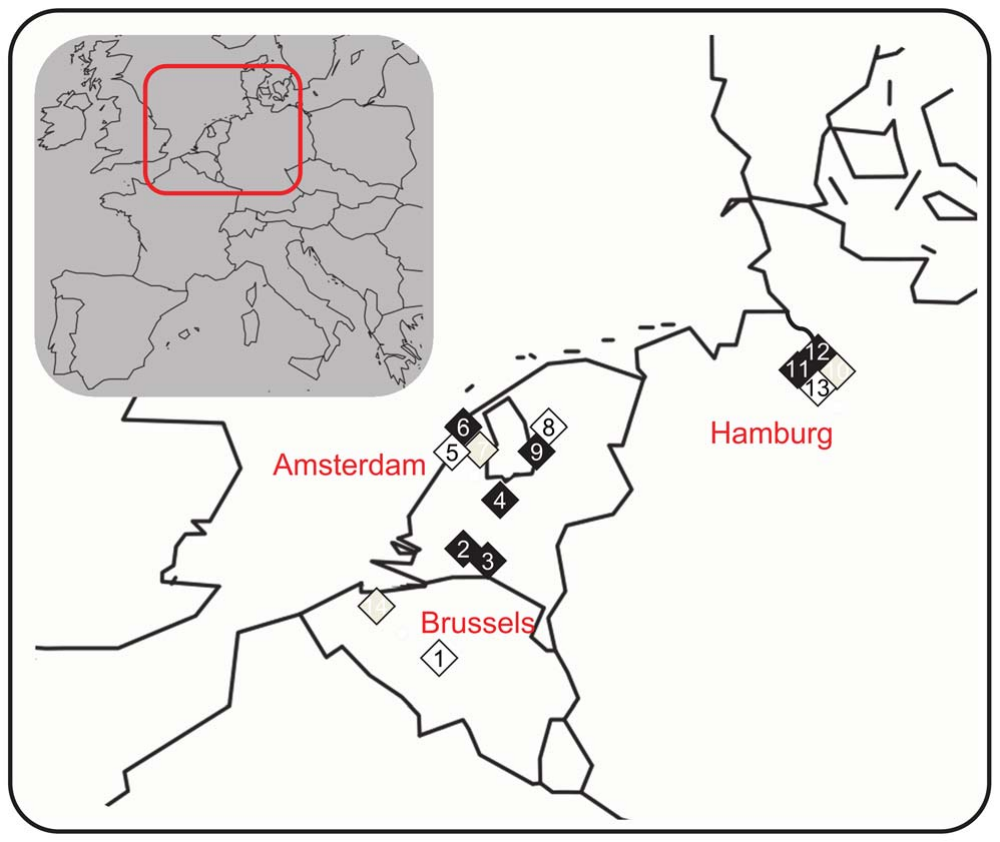
Location of 14 *L. stagnalis* populations involved in *Q*_ST_-*F*_ST_ comparisons. Populations are coded from 1 to 14. Colours indicate environmental categories (white, grey, and black, for GEP, GEP1, and GEP2, respectively). See Table 1 for details.

Adult individuals were collected from 14 natural populations during reproduction, in the course of a single campaign (5–12 July 2011) (Fig. 1). Sampling was conducted in public locations that did not require specific authorization, and did not involve endangered or protected species. Locality information and sampling characteristics are summarized in Table 1. Pesticide and other anthropogenic pressures were estimated from land-use patterns observed in the immediate surroundings of sampled sites, and using Google Earth. In the present study, we were not interested in an instant environmental concentration of pesticides at the time of sampling. On the contrary, under the tested hypothesis, we assumed historical contamination (repeated or chronic) as hypothetical selective force driving phenotypic evolution. Our strategy built on the use of landscape geographic information and agricultural land-use data. These have proved to be relevant tools to predict water body contamination by pesticides [51] and to assess pesticide exposure in the field (ditches [52]; ponds [18],[53],[54]). Moreover, in such aquatic systems, most variation may be explained by close land-use patterns (within a 100 m radius area), as demonstrated for water quality and vegetation complexity [55]. Consistently, we characterized each population's environment in terms of percent coverage by forest or moorland, pasture, crop (including potatoes, corn, orchards, bulb plants), and urban zone (Table 1). By crossing field observations and Google Earth satellite views with informations obtained from farmers on their practices, we estimated coverage proportions using the software *ImageJ* (http://rsbweb.nih.gov/ij/). When available, pesticide concentration data in surface water near the sampled sites were found to be globally consistent with our classification (http://81.93.58.66/bma_nieuw/begin.html; http://www.milieurapport.be/). Finally, populations were also classified according to four criteria: habitat (H: pond, channel, ditch), pesticide pressure (PP: two levels, low vs high), other anthropogenic pressure (OAP: two levels, low vs high), and global environmental pressure, as a combination of pesticide and other anthropogenic pressures (GEP: three levels).

**Table 1:** Geographical, environmental, and genetic characteristics of 14 *L. stagnalis* populations involved in F_ST_-Q_ST_ comparisons.

Localization	Code	Geographic coordinates	Land use	Genetic/Environmental factor					Population genetics						
				GC	H	PP	OAP	GEP	n	*N*	*A*_R_	*H*_E_	*F*_IS_	Selfing rate (%)	*Ne* [CI 95%]
CASTRICUM	CAS	52°33.17N	100% moor	West	Pond	0	0	0	32	3.3	2.60	0.341	0.327*	0.0	31
(Alkmaar, NL)	(5)	4°37.22E													[19]; [58]
KUINRE	KUI	52°47.67N	98% forest	West	Ditch	0	0	0	25	3.8	3.33	0.428	0.207*	0.0	25
(Nordoostpolder, NL)	(9)	5°47.69E	2% urban												[14]; [52]
HEDENDORF	HED	53°29.47N	90% forest	East	Pond	0	0	0	26	2.8	2.39	0.315	0.258*	0.0	32
(Hamburg, DE)	(13)	9°36.55E	10% urban												[18]; [66]
OUD-HEVERLEE	OUD	50°49.39N	55% forest	West	Pond	0	0	0	13	3.3	3.28	0.416	0.177	40.6*	26
(Brussels, BE)	(1)	4°39.29E	45% urban												[13]; [66]
SCHOORLDAM	SCH	52°43.13N	95% pasture	West	Channel	0	1	1	26	3.3	2.89	0.324	0.225*	18.5	24
(Alkmaar, NL)	(7)	4°42.2E	5% urban												[13]; [47]
DETSELBERGEN	DET	51°03.29N	30% forest	West	Pond	0	1	1	14	2.5	2.45	0.325	0.353*	48.5*	33
(Gent, BE)	(14)	3°49.35E	30% fallow												[16; 139]
			40% urban												
BUXTEHUDE	BUX	53°29.63N	80% fallow	East	Channel	0	1	1	27	3.2	2.84	0.355	0.094	0.0	22
(Hamburg, DE)	(10)	9°42.78E	15% crop												[12]; [43]
			5% urban												
BAARN	BAA	52°13.38N	25% pasture	West	Ditch	1	1	2	22	3.9	3.54	0.448	0.308*	12.0	33
(Utrecht, NL)	(4)	5°18.53E	25% crop												[18]; [72]
			50% urban												
OOSTEIND	OOS	51°38.87	30% pasture	West	Channel	1	1	2	32	4.8	4.18	0.590	0.175*	0.0	198
(Breda, NL)	(2)	4°55.82E	40% crop												[105; 698]
			30% urban												
BIEZENMORTEL	BIE	51°38.03N	20% forest	West	Channel	1	1	2	30	4.4	3.67	0.452	0.168*	9.1	109
(Breda, NL)	(3)	5°9.53E	5% pasture												[64; 259]
			75% crop												
PUTTEN	PUT	52°45.81N	30% crop	West	Ditch	1	1	2	32	2.9	2.62	0.370	0.216*	18.6	37
(Alkmaar, NL)	(6)	4°39.82E	70% urban												[22]; [67]
EMMELOORD	EMM	52°46.27N	80% crop	West	Ditch	1	1	2	24	3.7	3.32	0.456	0.252*	0.0	36
(Nordoostpolder, NL)	(8)	5°48.26E	20% urban												[20]; [80]
KOENIGSREICH	KOE	53°31.6N	85% crop	East	Channel	1	1	2	27	5.3	4.17	0.444	0.176*	9.5	48
(Hamburg, DE)	(11)	9°46.29E	15% urban												[29]; [91]
AGATHENBURG	AGA	53°34.54N	100% crop	East	Ditch	1	1	2	25	4.3	3.69	0.491	0.266*	30.7*	29
(Hamburg, DE)	(12)	9°32.82E													[17]; [53]

n indicates sample size, N mean number of alleles, A_R_ allelic richness, H_E_ expected heterozygosity, F_IS_ inbreeding coefficient, and Ne effective population size. Values significantly different from zero are indicated with an asterisk. GC holds for Genetic cluster and H for Habitat. Environmental pressure is described qualitatively under three alternative factors (PP: Pesticide pressure, OAP: Other Anthropogenic pressure, GEP: Global Environmental Pressure), the levels of which are defined as a function of land use within a radius of 100 m around sample sites (see text for details).

### Molecular analyses

DNA was chelex extracted from haemolymph or foot tissue from 399 wild-caught adults (14 to 33 snails per population). Neutral genetic variation was assessed at 12 microsatellite loci, i.e., A2, A112, B117 [56], 2k11 and 2k27 (Genbank accession: EF208747-EF208748 [57]), and EMLS04, EMLS13, EMLS21, EMLS26, EMLS29, EMLS41, EMLS45 [58], following the protocol described in Besnard et al. [58]. Only individuals with less than three missing genotypes were retained for population genetics analysis.

### Population neutral genetic structure

Mean allele number (*N*), allelic richness (*A_R_*), expected heterozygosity *H*_E_, and observed heterozygosity *H*_O_, were calculated with Genetix 4.05.2 [59]. The distribution of neutral genetic diversity within and among populations was estimated from Weir and Cockerham's estimators of Wright's *F* indices [60] using Fstat 2.9.3.2 [61]. Departures from HWE (heterozygote excess or deficiency) and linkage disequilibria were tested using Genepop 4.0.10 [62]. Population differentiation was tested with a permutation test, in which genotypes were permuted among samples (not assuming HWE within samples; see [61]). As *L. stagnalis* is a self-fertile hermaphroditic organism, the selfing rate was estimated per population and statistically compared to zero using Rmes [63]. Effective population size was estimated using the sibship assignment method, as implemented in the software Colony 2.0.3.0 [64] and assuming inbreeding, male and female polygamic mating systems, and monoecy.

To estimate the number of genetic clusters in our dataset without taking into account any predefined population, we used Structure 2.2 [65]. Analyses were performed assuming an admixture model and a number of genetic clusters (k) from 1 to 15. Each run started with a burn-in period of 50 000 steps followed by 300 000 Markov Chain Monte Carlo (MCMC) replicates. The most likely number of clusters was determined using the Δk statistic [66] using Structure Harvester [67]. We used Distruct to plot Structure output data [68].

The effect of environmental factors PP, OAP, GEP, H, and genetic cluster was assessed on population genetics parameters (*A_R_*, *H*_E_, *F*_IS_, *F*_ST_) with a permutation test using Fstat.

### Common garden experiment

**Figure 2:**
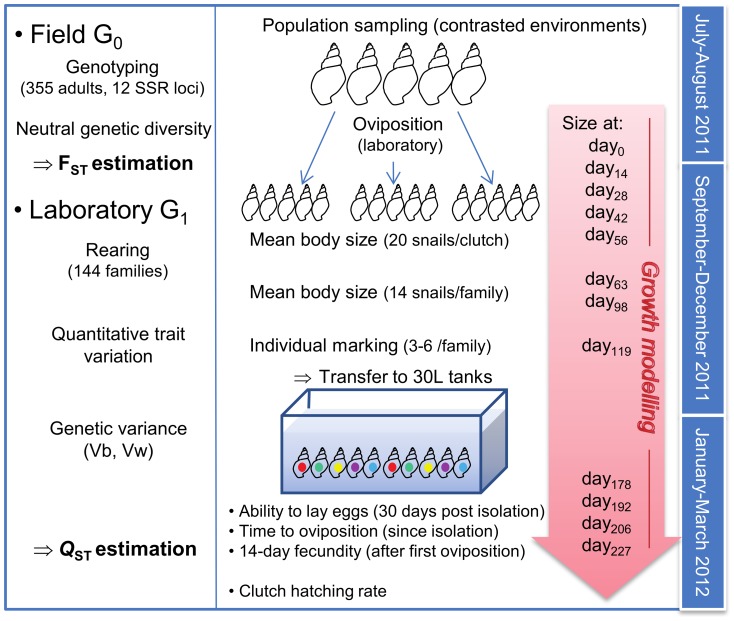
Schematic overview of the common garden experiment used to investigate population divergence in *L. stagnalis*.

Wild-caught adults were brought to the laboratory and reared under standard conditions at the INRA Experimental Unit U3E (Rennes, France), as previously described [69]. Snails were isolated in plastic vessels filled with 1 L de-chlorinated and charcoal filtered tapwater. They were fed weekly with 1.5 g of organic salad, at each water renewal. Room temperature was maintained at 20±1°C and the photoperiod was 16L/8D. Like other basommatophorans, *L. stagnalis* lays eggs embedded in mucous enveloppes which are deposited and fixed on available substrates (sediment, vegetation, tank walls, etc.). For a given snail, reproduction was followed during 14 days after the first clutch laid in the laboratory. A total of 228 snails over 399 reproduced after 3 months (57%). Families were characterized at various life-history traits (individual growth, female reproduction, hatching success), measured on the laboratory-born progeny (G_1_) as illustrated on Fig. 2. G_1_ snails were reared at 20±1°C, under a 14L/10D photoperiod.

### G_1_ rearing conditions

From hatching to the age of 119 days (roughly corresponding to the end of juvenile growth), G_1_ snails were reared as groups reflecting clutch origin (n = 427; 1 to 2 clutches per maternal parent). As individuals grew, rearing conditions were adjusted in terms of water volume, individual density, and food supply (see Table S1). Vessels were regularly moved in a randomized way. From the age of 119 days, 7 individuals per clutch were randomly chosen and marked with a honey bee mark (model FC075; diameter: 2.3 mm, weight: 1.8 mg; Ickowicz Apiculture, Bollene, France), and further reared in 30 L tanks (80 individuals per tank), in which they were fed twice a week with organic salad (0.5 g per snail), and from which 2/3 of water was renewed weekly.

### Life history traits: individual growth

From hatching to the age of 56 days, individual size (as inferred from shell height) was measured every two weeks on four randomly chosen juveniles per group. From the age of 63 days, all reared snails were individually measured. Measurements were performed with a stereomicroscope fitted with an ocular micrometer until day_63_, and a digital calliper afterwards. From the age of 119 days, three G_1_ snails per family (n = 492) were individually followed for growth and reproduction. For these 492 snails, size was measured at the age of 177.71±5.41 days, 191.71±5.41 days, 205.7±5.40 days, and 226.51±5.56 days (owing to variation in clutch age, and because measurements were performed at fixed dates after the age of 119 days), leading to a total of 12 values per individual. Growth was modelled using the Gompertz's model:
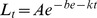
where *L_t_* is the shell height at time *t*, *A* the asymptotic shell length, *b* a scaling factor related to shell height at t = 0, and *k* reflects the growth rate. Using a preliminary subset of 20 individuals, this model performed better than the von Bertalanffy model (lower AIC), and was thus preferred for further growth analysis. Growth parameters were compared among families and populations, as well as size at hatching and size at 119 days.

### Life history traits: reproduction

Once clutches started to be observed in all aquaria, the 492 snails followed for growth were isolated in 200 mL plastic vessels and fed weekly with 1 g of organic salad immediately after water renewal. Several female reproductive traits were measured: time to first oviposition under isolation (with a censoring limit of 30 days), ability to lay eggs (as the proportion of reproductive snails per family), number of clutches and eggs laid during two weeks, clutch size (number of eggs per clutch), and clutch hatching rate.

### Statistical analyses

All traits were analyzed with generalized linear mixed effect models (GLMM, R-package *lme4*) [70], with appropriate error distribution (Gaussian for normal data, Poisson for count data, and binomial for proportions). When necessary, data were log- or BoxCox-transformed, and covariates were included in the model (see Table S2). The model structure was: *Y ∼ factor1 + factor2 + factor3 + covariate1 + covariate2 +…+ (1|population/family)*.

Fixed effects were tested by model comparison using a log-likelihood ratio test. Fixed factors were: genetic cluster (see Structure analysis), habitat, and environmental pressure (either pesticide, other anthropogenic, or global pressure). Family was nested within population, and both were treated as random factors. Global tests were followed by post-hoc pairwise comparison tests (Tukey and non parametric equivalent). All statistical analyses were performed using R 2.14.0 (R Core Team 2012).

### Q_ST_ -F_ST_ analysis

*Q*_ST_ was computed for each trait using the equation [71]; [72]:
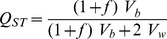



Where *f* is the inbreeding coefficient, *V_b_* the between population genetic variance and *V_w_* the within population genetic variance. *V_b_* was obtained directly from the population variance component, while for *V_w_*, as only the maternal origin could be assessed, family variance was used under the full-sib design (2*V_f_*) [73]. Within- and between-population variance components were estimated for each of the 11 studied traits using restricted maximum likelihood (REML) under a simplified mixed model (*Y∼1+ (1| pop/fam)*).

Population quantitative divergence was tested using the method of Whitlock and Guillaume [74], which accounts for variation among neutral loci used to estimate *F*_ST_, and for sampling error and variation in evolutionary history in *Q*_ST_ estimation. The method has lower type 1 error rate and better statistical power than previous tests, and seems particularly suited when neutral differentiation is high [46], as in *L. stagnalis* [58],[75]. Briefly, a bootstrapping procedure is applied to compare observed values of the difference *Q*_ST_ - *F*_ST_ to the *Q*_ST_ - *F*_ST_ distribution expected under the neutral hypothesis, as derived from *F*_ST_ distribution and variance components [60] and *Q*_ST_ neutral distribution predicted from the Lewontin-Krakauer distribution. Observed values were considered significant when they fell outside the 95% confidence interval of the neutral distribution [74].

## Results

### Population genetics

On 399 individual genotypes, 44 were discarded because of missing or unreadable data at more than three loci. Overall, the observed number of alleles per locus varied from three to 29, mean allelic richness ranged from 1.1 to 9.6 (Table S3), and genetic diversity per locus and sample varied from 0 to 0.86. Significant genotypic linkage disequilibrium was observed in 40 out of 656 comparisons. However, after Bonferroni correction, none of these remained significant. *F*_IS_-values varied widely across loci and samples, ranging from −0.238 (EMSL41 in BUX) to 1.000 (2k27 and B117 in DET; EMLS13 in HED; EMLS21 in BAA) (Table S3). Genetic parameters estimated per sample over loci reflected discrepancies between populations (Table 1). All populations except BUX and OUD were found significantly inbred (mean *F*_IS_-value  = 0.219, 95% CI [0.147; 0.287]). The selfing rate was significantly different from zero in three populations (OUD, DET, and AGA). Effective population size *Ne* was significantly larger in sites exposed to high pesticide pressure (PP1> PP0; Kruskal-Wallis test, *P* = 0.007).

**Figure 3:**
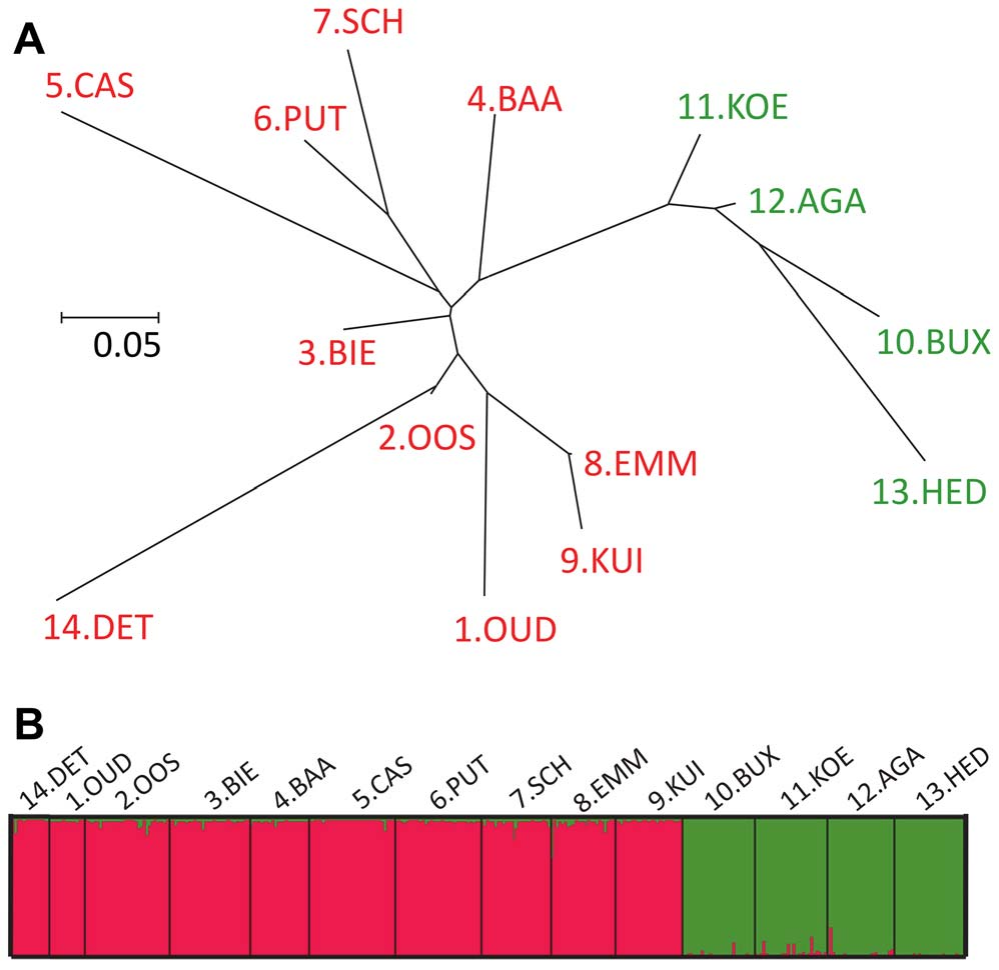
Population genetic differentiation in *L. stagnalis*. (A) Bayesian assignment probabilities in Structure analysis, for k = 2 clusters. Each bar represents an individual. Bar colour indicates the posterior probability that the individual belongs to the cluster of that color. (B) Unrooted Neighbour-Joining tree based on population pairwise *F*_ST_ values (see Table S4).

Population differentiation was generally high (mean *F_ST_*-value  = 0.291, 95% CI [0.248; 0.334]), as also reflected by pairwise estimates, which were all significant (Table S4), except between the two geographically close populations KUI and EMM. Interestingly, the four pond populations had the longest branches on the NJ-tree, indicating stronger differentiation associated with this habitat (Fig. 3A). From Structure analysis, individual genotypes clustered into two groups with the highest likelihood, and this was confirmed by the Δk statistic (Fig. 3B). Clusters corresponded to two geographic regions (10 western vs. four eastern populations). Therefore, the effect of “genetic cluster” was tested on life-history traits. No difference in level of genetic diversity, population inbreeding or population differentiation was found between the two clusters (Table 2).

**Table 2:** Comparison of *L. stagnalis* population genetic parameters as a function of different grouping factors.

Population grouping	*A*_R_	*H*_E_	*F*_IS_	*F*_ST_
GEP0	2.898	0.367	0.249	0.431
GEP1	2.725	0.353	0.190	0.450
GEP2	3.599	0.468	0.214	0.197
*P (GEP)*	*0.073*	*0.094*	*0.487*	***0.016***
PP0	2.824	0.362	0.226	0.413
PP1	3.599	0.468	0.214	0.197
*P(PP)*	***0.007***	***0.008***	*0.773*	***0.001***
OAP0	2.898	0.367	0.249	0.431
OAP1	3.337	0.438	0.210	0.248
*P(OAP)*	*0.219*	*0.178*	*0.329*	***0.031***
H Pond	2.678	0.343	0.282	0.502
H Ditch	3.300	0.436	0.246	0.221
H Channel	3.550	0.449	0.166	0.246
*P(H)*	*0.062*	*0.095*	***0.016***	***0.001***
H Pond	2.678	0.343	0.282	0.502
H Channel+Ditch	3.425	0.443	0.203	0.227
*P (P vs D+C)*	***0.025***	***0.038***	***0.034***	***0.002***
H Pond+Ditch	3.024	0.398	0.258	0.336
H Channel	3.550	0.449	0.166	0.246
*P (P+D vs C)*	*0.116*	*0.306*	***0.009***	*0.271*
H Pond+Channel	3.162	0.409	0.203	0.330
H Ditch	3.300	0.436	0.246	0.221
*P (P+C vs D)*	*0.686*	*0.641*	*0.285*	*0.178*
GC West	3.187	0.422	0.229	0.244
GC East	3.272	0.410	0.196	0.162
*P (GC)*	*0.827*	*0.837*	*0.426*	*0.393*

*A*_R_ is allelic richness, *H*_E_ expected heterozygosity, *F*_IS_ inbreeding coefficient, and *F*_ST_ differentiation index.

GEP holds for Global environmental pressure, PP for Pesticide pressure, OAP for Other anthropogenic pressure, H for Habitat (P =  Pond, D =  Ditch, C =  Channel), GC for Genetic cluster. Permutation-based statistical test (P value, 1000 permutations).

Population genetic parameters were affected by most environmental factors (Table 2). Genetic diversity tended to be greater under higher environmental pressure (GEP2, PP1, OAP1), although the difference was significant under pesticide pressure only (*A*_R_, P = 0.007; *H*_E_, P = 0.008). Population differentiation was significantly affected by global environmental pressure (GEP, P = 0.016) with a lower value among populations exposed to the highest level of pressure. With regard to specific pressures, lower differentiation was associated with pesticide as well as with other anthropogenic pressures (PP, P = 0.001; OAP, P = 0.031). Habitat affected all tested parameters, when ponds were compared to ditch and channel populations: pond populations were significantly less variable (*A*_R_, P = 0.025; *H*_E_, P = 0.038), more inbred (F_IS_, P = 0.034) and much more differentiated than were other populations (F_ST(pond)_ = 0.502 vs. F_ST(ditch + channel)_ = 0.227, P = 0.002).

### Life history variation

Most G_1_ traits showed significant heterogeneity at both population and family levels (Tables S2 and S5). No effect of genetic cluster was detected, excepted on early size, which was larger in eastern populations (size at hatching, P = 0.019; growth parameter *b*, P = 0.005; Table 3). Among environmental factors, habitat was the most effective, with six traits or parameters significantly different between systems. Growth parameters indicated that body size at t_0_ increased significantly from ponds to channels and from channels to ditches, whereas asymptotic size was larger in ponds than in channels and ditches (Fig. S1). With respect to reproduction, snails from ponds had a significantly lower reproductive activity than those from ditches and channels: lower ability to lay eggs, and in reproductive snails, longer time to oviposition. Clutch size decreased significantly from ponds to ditches and from ditches to channels, and clutch hatching rate was lower in pond and ditch populations than in channel ones.

**Table 3:** Summary statistics of fixed effects tested on *L. stagnalis* life history traits.

Trait/parameter	Genetic cluster (W/E)		Habitat (P/C/D)		Pesticide pressure (PP0/1)		Other anthropogenic pressure (OAP0/1)		Global environmental pressure (GP0/1/2)	
	χ^2^_df = 1_	P	χ^2^_df = 2_	P	χ^2^_df = 1_	P	χ^2^_df = 1_	P	χ^2^_df = 2_	P
*Growth*										
Size at hatching	**6.95**	**0.008**	3.67	0.159	0.12	0.724	0.36	0.549	0.91	0.636
	*W<E*									
Growth parameter *b*	**9.23**	**0.002**	**17.91**	**<0.001**	0.44	0.506	**9.60**	**0.002**	**10.20**	**0.006**
	*W>E*		*P>C>D*				*OAP0>OAP1*		*GP0 = GP1>GP2*	
Growth parameter *k*	1.76	0.185	0.43	0.805	0.66	0.415	0.04	0.835	1.10	0.576
Growth parameter *A*	0.25	0.614	**8.21**	**0.016**	0.010	0.919	3.01	0.083	3.68	0.159
			*P>C = D*				*OAP0>OAP1*			
Size at 119 days	1.83	0.176	2.06	0.358	0.00	0.955	0.11	0.746	0.12	0.944
*Reproduction*										
Ability to lay eggs	0.11	0.745	**9.45**	**0.009**	3.30	0.069	1.75	0.186	3.96	0.138
			*P<D = C*		PP0<PP1					
Time to oviposition	2.42	0.200	4.92	0.085	0.15	0.699	1.08	0.300	1.08	0.582
			*P>D = C*							
Number of clutches	1.40	0.237	0.39	0.823	0.14	0.708	0.10	0.756	0.44	0.802
Number of eggs	0.55	0.457	4.96	0.084	0.21	0.646	0.69	0.405	1.65	0.439
			*P = D<C*							
Clutch size	2.55	0.110	**11.18**	**0.004**	0.06	0.807	1.93	0.165	3.11	0.212
			*P<D<C*							
Hatching rate	0.59	0.441	**6.41**	**0.041**	0.98	0.322	**6.25**	**0.012**	**6.25**	**0.044**
			*P = D<C*				*OAP0>OAP1*		*GP0>GP1 = GP2*	

General model: glmer  =  Y _∼_ covar + Genetic cluster + Habitat + Environmental pressure + (1 | pop/fam).

Growth parameters: A is asymptotic size, b is related to size at birth, k to growth rate. *Bold values are statistically significant.*

Compared to aquatic system, human pressure appeared much less effective. First, pesticide pressure had no effect, except a marginal one on ability to lay eggs, found greater in exposed populations (P = 0.069). Second, other anthropogenic pressures affected growth parameter b (significantly larger size at t_0_ under OAP1) and hatching rate (significantly lower under OAP1) (Fig. S2). Interestingly, lower hatching rates were already observed on the clutches laid by G_0_ snails inhabiting OAP1 sites (720 clutches, GLMM test: χ^2^ = 11.861, P*<*0.001). Third, global pressure affected *b* (significantly larger snails at t_0_ under GEP2), and hatching rate, which was significantly impaired in exposed populations (GEP1 and GEP2) relative to reference ones (GEP0).

**Figure 4:**
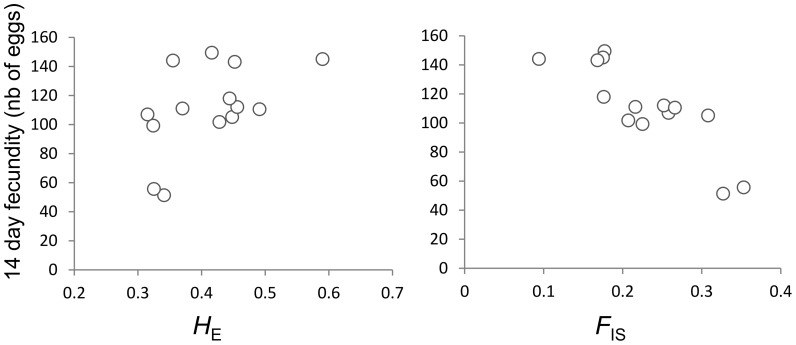
Correlation plot between 14-days fecundity and (A) population expected heterozygosity, (B) population inbreeding, as based on 14 *L. stagnalis* populations.

Several life history traits correlated with population genetic characteristics. First, fecundity was positively correlated with *H*_E_ (r = 0.524, P = 0.031; Fig. 4A), and with *A*_R_ (r = 0. 489, P = 0.044). Second, a negative correlation was found between population inbreeding and G_1_ fecundity (r = −0.845, P<0.001; Fig. 4B), ability to reproduce (r = −0.664, P = 0.006), and clutch hatching rate (r = −0.634, P = 0.009) (see Table 1 and Table S5).

### Q_ST_-F_ST_ analysis

Results are summarized on Table 4. Over the set of 11 studied traits or parameters, four traits were found to evolve consistently with neutral expectations (hatching size, growth parameter *k*, number of eggs, clutch size), whereas homogenizing selection was indicated for two traits (growth parameter *b*, clutch hatching rate), and divergent selection for five traits (size at 119 days, asymptotic size *A*, ability to lay eggs, time to oviposition, number of clutches laid).

**Table 4:** Summary of *Q*_ST_ *-F*_ST_ analyses on laboratory G1s of 14 *L. stagnalis* populations.

Trait/Parameter	N	N*fam*	V*_b_*	V*_f_*	Observed *Q*_ST_ - *F*_ST_	Neutral *Q*_ST_-*F*_ST_ 95% CI	P-value left	P-value right	Divergence pattern
*Growth*									
Hatching size	427	211	1.46E-03	1.86E-03	−0.098	[−0.160; 0.150]	0.141	0.859	Neutrality
Parameter *b*	492	144	4.52E-04	1.19E-03	−0.187	[−0.160; 0.154]	0.009	0.991	Homogenizing selection
Parameter *k*	492	144	2.48E-07	2.99E-07	−0.089	[−0.155; 0.156]	0.169	0.831	Neutrality
Parameter *A*	492	144	6.02E-03	6.56E-04	0.446	[−0.153; 0.155]	1.000	0.000	Divergent selection
Size at 119 days	2855	183	2.26E+01	5.07E+00	0.285	[−0.151; 0.155]	1.000	0.000	Divergent selection
*Reproduction*									
Ability to lay eggs	492	144	2.70E+00	5.39E-08	0.709	[−0.153; 0.157]	1.000	0.000	Divergent selection
Time to oviposition	492	144	2.86E-02	8.36E-03	0.219	[−0.141; 0.151]	0.996	0.004	Divergent selection
Number of clutches	492	144	1.10E-01	1.02E-10	0.709	[−0.160; 0.151]	1.000	0.000	Divergent selection
Number of eggs	492	144	8.22E+02	5.64E+02	0.017	[−0.162; 0.163]	0.605	0.395	Neutrality
Clutch size	1537	144	2.95E+00	1.47E+00	0.088	[−0.158; 0.168]	0.885	0.115	Neutrality
Hatching rate	1537	144	3.27E-01	6.29E-01	−0.154	[−0.157; 0.160]	0.025	0.975	Homogenizing selection

Analyses were performed using a full-sib design and accounting for population inbreeding.

N =  sample size, Nfam  =  number of families, Vb and Vf indicate between population and family variance, respectively. Gompertz growth parameters: A is asymptotic size, b is related to size at birth, k is related to growth rate. Left and right P-values indicate the probability for Q_ST_-F_ST_ observed value to lie within the neutral distribution (unilateral test based on 1000 bootstraps, α = 0.025).

## Discussion

The hypothesis that human activities may induce population adaptive divergence in *L. stagnalis* was addressed under a common garden experiment involving life history traits and a set of natural populations from contrasted environments. Five traits or parameters over 11 were found under divergent selection, despite a strong neutral genetic structure. The occurrence of two genetic clusters (Eastern and Western populations) had only a weak influence on quantitative genetic divergence, suggesting that population level was the adequate scale to draw selection inferences [76]. Only size at hatching and its corresponding growth parameter (*b*) were affected by this factor (larger hatchlings to the East). Given that early size was found under uniform selection, it was assumed that the genetic divergence associated to geography did not interfere with selection putatively associated with local environmental pressures.

### Selection versus random genetic drift

All traits found to diverge adaptively were adult traits, related to late expressed growth (sub-adult size, asymptotic size *A*) and to reproduction (ability to reproduce, time to oviposition after isolation, number of clutches). Conversely, uniform selection was the most likely evolutionary hypothesis for early survival and size (hatching success and growth parameter *b*, which is related to size at t_0_). Globally, inferred patterns, i.e., early (late) traits under homogenizing (divergent) selection, are in lines with those found in another freshwater snail [47],[77].

Traditional statistical analyses performed on the G_1_ revealed that, to the exception of a marginal effect of pesticide pressure on ability to lay eggs (P = 0.069), habitat was the only factor affecting most traits found under divergent selection. Indeed, pond populations were characterized by larger ultimate size (growth parameter *A*), lower ability to lay eggs, and longer time to oviposition after isolation, compared to ditch and channel ones.

Congruent with *L. stagnalis* low dispersion and weak habitat connectivity, pond populations were particularly differentiated (especially CAS and DET, pairwise *F*_ST_) and presented generally lower genetic diversity and higher inbreeding than channel and ditch populations. Moreover, compared to channel populations, pond and ditch populations were also more inbred (P = 0.009). These aquatic systems are likely to imply stronger population isolation, even among ditches (due to frequent drought). A similar influence of habitat was emphasized in another freshwater snail, *Physa acuta* [78]. Thus, in *L. stagnalis*, although divergent selection was detected for traits also affected by habitat type, genetic drift is likely to highly contribute to divergence among habitats. This result is in line with the detection of significant drift load in experimental populations occupying outdoor close mesocosms [69].

In the common frog *Rana temporaria*, habitat fragmentation correlates with low genetic diversity and high differentiation, and negatively impacts tadpole body size [79], which is a critical trait determining individual fitness [80]. In *L. stagnalis*, although ultimate body size was greater in ponds, the relation between size and fitness is not necessarily positive, because of possible energetic trade-offs between growth and reproduction [81]. Indeed, two pond populations (CAS and DET) exhibited both highest mean asymptotic size and lowest fecundity (see Table S5).

The *Q*_ST_-*F*_ST_ based test concluded to neutral divergence for size at hatching, growth rate (parameter *k*), and two reproductive traits, clutch size and early fecundity (number of eggs laid during 14 days following first clutch since isolation). On the latter, it seems surprising to find no hint of selection, either uniform or divergent, although this may simply reflect low heritability of fitness traits [82]. In support of this, it is worth noting that fecundity is extremely high in *L. stagnalis* (from adulthood to death, i.e., about one year under standard conditions, a snail produces more than 10 progeny per day), suggesting the possibility of high variation without drastic fitness consequences. Consistently, population growth rate was found to be insensitive to fecundity under experimental conditions (Leslie matrix modelling [83]). Alternatively, apparent neutral evolution might result from a bias in *Q*_ST_ estimation. Under genetic drift, dominance and epistasis bias *Q*_ST_ estimates downward [84]–[86]. As *Q*_ST_ is traditionally estimated without accounting for these non-additive sources of variation, and given the strong observed global *F*_ST_ value, divergent selection might have been underestimated for fecundity. On the other side, a hypothetical upward bias in *Q*_ST_ estimation might result from too high a mutation rate at the markers used to infer *F*_ST_, as compared to the mutation rate of loci encoding the studied quantitative trait [87],[88]. This hypothesis is invalidated by observed *F*_ST_ values, which were rather high and significant, although they may also suffer from a downward bias. Finally, gene flow may well lead to an apparent pattern of adaptive divergence at a trait under uniform selection, if this trait is correlated to a trait under divergent selection [89]. Again, high population neutral differentiation makes this hypothesis unlikely. Besides methodological causes of bias, it might be also mentioned that (1) true lifespan fecundity was not measured, and (2) as a source of uncontrolled variation, no distinction could be made between outcrossing and potential selfing in the G_1_.

As reported above, homogenizing selection was indicated for early traits, which is expected to reflect the occurrence of a uniform fitness optimum between the studied populations. *Q*_ST_ estimation was based on the G_1_ of wild-caught adult snails, with the exception of survival at hatching (measured on G_2_). G_1_ families correspond hence to the hermaphroditic equivalent of isofemale lines, as used for broad-sense heritability estimation [73]. Therefore, these “broad-sense” *Q*_ST_ estimates are potentially biased by genetic non additive sources of variation (dominance, epistasis), and by maternal effects. The latter are likely to operate early in life and tend to recede with age [90]. Hence, in the present study, traits found under uniform selection (early expressed traits) might be more prone to such a bias than traits found under divergent selection (late expressed traits). However, as complex traits seem to vary mostly additively [91],[92], we hypothesized, as done in most *Q*_ST_-*F*_ST_ comparisons [46], that neglecting these sources of variation would have little consequences.

Alternatively to the uniform selection hypothesis, the pattern exhibited by early traits would result from trait canalization, as a phylogenetically inherited characteristics [86]. Since the pattern was similar to that observed in *Galba truncatula* [77], the criterion of cross-species consistency (supporting the canalization hypothesis) may be met in basommatophorans, but this would need to be confirmed in other species. Further investigation is clearly needed to disentangle both causes of *L. stagnalis* population phenotypic convergence.

### Genetic diversity and fitness

The positive correlation observed between population genetic variability and G_1_ fecundity, is consistent with previous findings in *L. stagnalis* [93]. Under the assumption that G1 performances reflect the fitness of natural populations, the present result also supports the hypothesis that local drift load can be strong in this species, as previously suggested [69]. Therefore, under high random genetic drift, as indicated here, it might also be asked to what extent *Q*_ST_ is modified by the expected stochastic evolution of slightly deleterious alleles as compared to pure selection-based predictions (random drift load [37]).

Furthermore, the negative correlation observed between population inbreeding and several traits related to fecundity (ability to reproduce, time to oviposition, early fecundity, and hatching rate) is in line with the occurrence of reduced fitness in inbred populations, and thus of inbreeding depression in these populations. However, as inbreeding was found significant in most populations and because effective population size was usually small, it is suggested that random genetic drift is actually the main cause of these apparent correlations, through reduced genetic diversity and drift load accumulation caused by population isolation and small size [32]. This hypothesis is still strengthened by the fact that population inbreeding correlated negatively with G_2_ survival at hatching (i.e., irrespective of individual inbreeding level). Inbreeding depression estimated under selfing relative to random outcrossing (ID) is particularly low in *L. stagnalis* [69],[93]–[95], despite a clear preference for outcrossing [96],[97]. Thus, although ID was not estimated, it seems unlikely that selfing could be responsible for the observed pattern. In support of this, no correlation was observed between population selfing rate and G_1_ fecundity.

### Evolutionary impact of anthropogenic pressures

Neutral genetic variation was inflated in populations exposed to anthropogenic pressures, including pesticides. This was reflected in terms of genetic diversity, allelic richness, and effective population size, and through lower genetic differentiation. These results are opposed to those found in experimental populations of *L. stagnalis* exposed to cocktails of pesticides [98]. More generally, they are not consistent with the hypothesis of increased local stochasticity due to anthropogenic pressure (see introduction). High genetic diversity may result either from the maintenance of large population size despite stressful conditions, or from significant gene flow (immigration). Alternatively, undetected population subdivision may also be responsible for the observed patterns, as subdivision is expected to maintain diversity at the global scale. Furthermore, at the same scale, heterozygote deficiencies are also to be expected (Wahlund effect). Therefore, population subdivision into local demes might be responsible for part of the within population fixation observed in the dataset. As a possible impact of anthropogenic pressure, this potential effect should deserve specific attention, e.g., through the scoring of individual location at the time of sampling, and using assignment tests. As already mentioned, the effect of anthropogenic pressures on life history traits was much lower than that of habitat. This was particularly marked for pesticide pressure, which was the primary focus of the study. Three possible causes for this may be discussed: 1) true lack of pesticide effect, 2) effects underestimated due to confounding environmental factors, and 3) experimental conditions too benign to detect adaptive divergence.

Besides the hypothesis of pesticide innocuousness to gastropod populations, one explanation may be that sampling did not reflect the effective magnitude of pesticide pressure. However, as the design included areas of intensive agriculture, and due to the close proximity of water bodies and treated agricultural parcels, this seems unrealistic. Alternatively, evolutionary patterns associated with pesticide exposure may have been masked by other environmental characteristics, an idea supported by the significant habitat effect observed on most studied traits. Thus, although the number of populations and families was close to the recommended values [72], and despite the ability of mixed effect models to treat unbalanced designs [70], our experiment may lack sufficient statistical power, probably due to the lack of ponds within agricultural zones and of channels in reference sites. Meanwhile, the present study emphasizes the need to take habitat into account in *Q*_ST_-*F*_ST_ comparisons [79].

Third, the apparent lack of effect of pesticide pressure on the studied traits might also result from experimental conditions, which were too benign. For example, the trend for enhanced ability to reproduce observed in the G_1_ from polluted areas, might reflect a specific response of exposed populations to overcome early mortality induced by chemicals (hatching rate: OAP0>OAP1 in G_1_ and G_2_), which was unexpected to express under common garden conditions. In the context of evolution under stressful environments, trait expression would be best studied under a gradient of stress conditions, as also recommended for molecular traits and pathways [15]. Stressful conditions may indeed facilitate the detection of divergent selection patterns with respect to pesticide historical exposure, since *Q*_ST_ has been shown to depend on the experimental environment [48] [99].

Finally, the present study also emphasized the occurrence of high genetic variation both at neutral markers and fitness-related traits in a species used as model in ecotoxicology. This finding provides empirical support for the need to account for genetic variation in toxicity testing and, as a perspective, in future procedures of ecological risk assessment [12].

## Supporting Information

Figure S1 ***L. stagnalis*** **individual growth (G_1_ snails) according to population habitat.** Gompertz growth curves based on 12 dates of measurement, performed on 492 individuals.
(TIFF)

Figure S2 ***L. stagnalis*** **hatching rate as a function of anthropogenic pressure (OAP, n = 1537 clutches).**
(TIFF)

Table S1 Evolution of *L. stagnalis* G1 rearing conditions as a function of age.
(DOCX)

Table S2 Summary of statistical analyses performed on *L. stagnalis* life history traits.
(DOCX)

Table S3 Population genetic parameters calculated per locus and population on 14 *L. stagnalis* population samples (see materials and methods for explanation of population codes).
(DOCX)

Table S4 Population genetic differentiation estimated among 14 *L. stagnalis* populations, on the basis of 12 SSR loci.
(DOCX)

Table S5 Life-history trait/parameter mean-values (SE) estimated in the laboratory-born G_1_ from 14 *L. stagnalis* populations used to test patterns of adaptive divergence (*Q*_ST_-*F*_ST_ analysis).
(DOCX)
