# Supplementary material for: Environmental versus Anthropogenic Effects on Population Adaptive Divergence in the Freshwater Snail Lymnaea stagnalis
Source: PLoS One. 2014 Sep 10;9(9):e106670. doi: 10.1371/journal.pone.0106670 (PMC4160221; doi:10.1371/journal.pone.0106670)
Supplement: Table S1 — Evolution of L. stagnalis G1 rearing conditions as a function of age. (DOCX) [file pone.0106670.s003.docx]

**Table S1.** Evolution of *L. stagnalis* G1 rearing conditions as a function of age.

|  | Water volume | Group size | Food weekly supply (organic salad) |
| --- | --- | --- | --- |
| Hatching to day_42_ | 75 ml | 20 | 0.5 g |
| day_42_ to day_63_ | 75 ml | 20 | 1 g |
| day_63_ to day_98_ | 750 ml | 14 | 3 g |
| day_98_ to day_119_ | 750 ml | 14 | 6 g |
