# Supplementary material for: Environmental versus Anthropogenic Effects on Population Adaptive Divergence in the Freshwater Snail Lymnaea stagnalis
Source: PLoS One. 2014 Sep 10;9(9):e106670. doi: 10.1371/journal.pone.0106670 (PMC4160221; doi:10.1371/journal.pone.0106670)
Supplement: Table S2 — Summary of statistical analyses performed on L. stagnalis life history traits. (DOCX) [file pone.0106670.s004.docx]

**Table S2.** Summary of statistical analyses performed on *L. stagnalis* life history traits. General model (see Table 3 for fixed factor effects):

*glmer =Y _~_ covar + Genetic cluster + Habitat + Environmental pressure + (1 | pop / fam)*

|  |  | Random factors | |  | Covariates | | | | | |
| --- | --- | --- | --- | --- | --- | --- | --- | --- | --- | --- |
| G_1_ Traits |  | Pop | Family |  | G_0_ clutch size | Development time of G_0_ clutch | G_0_ size | 14_days_ fecundity | Growth during fecundity monitoring | Age at the start of fecundity monitoring |
| *Growth* |  |  |  |  |  |  |  |  |  |  |
| Hatching size | χ²_1d.f._ | **4.22** | **12.69** |  | **9.28** | **11.42** | 2.77 | - | - | - |
|  | P | **0.04** | **< 0.001** |  | **< 0.001** | **< 0.001** | 0.096 |  |  |  |
| Parameter *b* | χ²_1d.f._ | 0.18 | 2.06 |  | - | - | 0 | 1.72 | - | - |
|  | P | 0.67 | 0.151 |  |  |  | 1 | 0.19 |  |  |
| Parameter *k* | χ²_1d.f._ | 1.98 | **10.73** |  | - | - | 0 | **34.47** | - | - |
|  | P | 0.159 | **0.001** |  |  |  | 1 | **< 0.001** |  |  |
| Parameter *A* | χ²_1d.f._ | **23.4** | **2.92** |  | - | - | 0 | **12.94** | - | - |
|  | P | **< 0.001** | **0.087** |  |  |  | 1 | **< 0.001** |  |  |
| Size at 119 days | χ²_1d.f._ | **23.25** | 3.14 |  | - | - | **535.52** | - | - | - |
|  | P | **< 0.001** | 0.077 |  |  |  | **< 0.001** |  |  |  |
| *Reproduction* |  |  |  |  |  |  |  |  |  |  |
| Ability to lay eggs | χ²_1d.f._ | **6.72** | 3.00E-4 |  | - | - | - | - | **15.83** | 0 |
|  | P | **0.01** | 0.99 |  |  |  |  |  | **< 0.001** | 1 |
| Time to oviposition | χ²_1d.f._ | **28.32** | **11.38** |  | - | - | - | - | **73.36** | 0.12 |
|  | P | **< 0.001** | **< 0.001** |  |  |  |  |  | **< 0.001** | 0.731 |
| Number of clutches | χ²_1d.f._ | **32.47** | 0 |  | - | - | - | - | **15.17** | 0.16 |
|  | P | **< 0.001** | 1 |  |  |  |  |  | **< 0.001** | 0.693 |
| Number of eggs | χ²_1d.f._ | **7.18** | **12.71** |  | - | - | - | - | **58.03** | 0.11 |
|  | P | **0.007** | **< 0.001** |  |  |  |  |  | **< 0.001** | 0.74 |
| Clutch size | χ²_1d.f._ | **10.5** | **32.09** |  | - | - | - | - | **45.93** | **23.76** |
|  | P | **0.001** | **< 0.001** |  |  |  |  |  | **< 0.001** | **< 0.001** |
| Hatching rate | χ²_1d.f._ | **8.42** | **1188.2** |  | - | - | - | - | **176.54** | **5.68** |
|  | P | **0.004** | **< 0.001** |  |  |  |  |  | **< 0.001** | **0.017** |

*Statistically significant values in bold.*
