# Supplementary material for: Environmental versus Anthropogenic Effects on Population Adaptive Divergence in the Freshwater Snail Lymnaea stagnalis
Source: PLoS One. 2014 Sep 10;9(9):e106670. doi: 10.1371/journal.pone.0106670 (PMC4160221; doi:10.1371/journal.pone.0106670)
Supplement: Table S3 — Population genetic parameters calculated per locus and population on 14 L. stagnalis population samples (see materials and methods for explanation of population codes). (DOCX) [file pone.0106670.s005.docx]

**Table S3.** Population genetic parameters calculated per locus and population on 14 *L. stagnalis* population samples (see materials and methods for explanation of population codes).

| Locus | Overall | 1.OUD | 2.OOS | 3.BIE | 4.BAA | 5.CAS | 6.PUT | 7.SCH | 8.EMM | 9.KUI | 10.BUX | 11.KOE | 12.AGA | 13.HED | 14.DET |
| --- | --- | --- | --- | --- | --- | --- | --- | --- | --- | --- | --- | --- | --- | --- | --- |
| 2k11 |  |  |  |  |  |  |  |  |  |  |  |  |  |  |  |
| *N* | 336 | 13 | 31 | 30 | 21 | 30 | 32 | 25 | 23 | 24 | 25 | 25 | 19 | 26 | 12 |
| *Na* | 14 | 5 | 8 | 8 | 3 | 4 | 3 | 4 | 4 | 3 | 5 | 8 | 8 | 3 | 3 |
| *A*_R_ | 7.633 | 4.85 | 6.13 | 6.56 | 2.93 | 3.62 | 2.99 | 3.35 | 3.94 | 2.48 | 4.54 | 6.49 | 7.48 | 2.99 | 3.00 |
| *H*_o_ | 0.530 | 0.615 | 0.903 | 0.567 | 0.571 | 0.433 | 0.563 | 0.480 | 0.609 | 0.250 | 0.480 | 0.480 | 0.474 | 0.423 | 0.500 |
| *H*_e_ | 0.822 | 0.683 | 0.770 | 0.790 | 0.529 | 0.481 | 0.569 | 0.545 | 0.565 | 0.228 | 0.607 | 0.700 | 0.868 | 0.597 | 0.594 |
| *F*_IS_ | **0.356** | 0.103 | -0.176 | 0.286 | -0.084 | 0.1 | 0.012 | 0.121 | -0.079 | -0.1 | 0.212 | 0.319 | **0.461** | 0.295 | 0.165 |
| A112 |  |  |  |  |  |  |  |  |  |  |  |  |  |  |  |
| *N* | 349 | 13 | 32 | 29 | 19 | 32 | 31 | 26 | 24 | 25 | 27 | 27 | 25 | 26 | 13 |
| *Na* | 29 | 7 | 12 | 8 | 8 | 1 | 2 | 2 | 5 | 9 | 3 | 10 | 9 | 3 | 4 |
| *A*_R_ | 9.261 | 6.84 | 8.88 | 6.29 | 6.91 | 1.00 | 1.99 | 1.46 | 4.70 | 7.33 | 2.70 | 6.36 | 7.25 | 2.46 | 4.00 |
| *H*_o_ | 0.332 | 0.769 | 0.406 | 0.448 | 0.316 | 0.000 | 0.290 | 0.039 | 0.125 | 0.440 | 0.444 | 0.444 | 0.160 | 0.423 | 0.846 |
| *H*_e_ | 0.759 | 0.812 | 0.818 | 0.794 | 0.676 | 0.000 | 0.252 | 0.039 | 0.719 | 0.816 | 0.545 | 0.743 | 0.764 | 0.497 | 0.699 |
| *F*_IS_ | **0.562** | 0.055 | **0.507** | **0.44** | **0.539** | _ | -0.154 | 0 | **0.829** | **0.466** | 0.188 | **0.406** | **0.794** | 0.151 | -0.222 |
| 2k27 |  |  |  |  |  |  |  |  |  |  |  |  |  |  |  |
| *N* | 349 | 13 | 31 | 29 | 22 | 31 | 30 | 26 | 23 | 25 | 27 | 27 | 25 | 26 | 14 |
| *Na* | 5 | 4 | 4 | 3 | 3 | 4 | 4 | 3 | 3 | 3 | 4 | 3 | 3 | 3 | 2 |
| *A*_R_ | 3.919 | 3.85 | 3.97 | 2.96 | 2.99 | 2.88 | 3.04 | 2.96 | 2.90 | 2.73 | 3.14 | 2.84 | 2.94 | 2.70 | 2.00 |
| *H*_o_ | 0.338 | 0.385 | 0.581 | 0.483 | 0.318 | 0.032 | 0.200 | 0.308 | 0.391 | 0.360 | 0.333 | 0.519 | 0.480 | 0.231 | 0.000 |
| *H*_e_ | 0.664 | 0.345 | 0.698 | 0.402 | 0.555 | 0.211 | 0.403 | 0.456 | 0.484 | 0.411 | 0.486 | 0.469 | 0.581 | 0.275 | 0.349 |
| *F*_IS_ | **0.491** | -0.121 | 0.17 | -0.206 | 0.432 | **0.849** | 0.508 | 0.33 | 0.195 | 0.126 | 0.318 | -0.108 | 0.177 | 0.162 | 1 |
| A2 |  |  |  |  |  |  |  |  |  |  |  |  |  |  |  |
| *N* | 345 | 12 | 31 | 30 | 21 | 32 | 29 | 24 | 23 | 25 | 27 | 26 | 25 | 26 | 14 |
| *Na* | 14 | 5 | 7 | 11 | 7 | 9 | 5 | 6 | 7 | 7 | 6 | 9 | 7 | 4 | 4 |
| *A*_R_ | 7.914 | 5.00 | 6.68 | 7.79 | 6.31 | 4.86 | 3.83 | 4.51 | 5.70 | 4.91 | 4.86 | 7.37 | 5.56 | 3.45 | 3.86 |
| *H*_o_ | 0.481 | 0.417 | 0.581 | 0.633 | 0.524 | 0.344 | 0.207 | 0.500 | 0.478 | 0.480 | 0.630 | 0.808 | 0.440 | 0.423 | 0.071 |
| *H*_e_ | 0.817 | 0.801 | 0.820 | 0.682 | 0.797 | 0.464 | 0.698 | 0.582 | 0.627 | 0.629 | 0.699 | 0.789 | 0.692 | 0.635 | 0.516 |
| *F*_IS_ | **0.411** | 0.491 | 0.295 | 0.072 | 0.348 | 0.262 | **0.707** | 0.144 | 0.241 | 0.24 | 0.101 | -0.024 | 0.369 | 0.338 | **0.866** |

**Table S3.** *continued.*

| B117 |  |  |  |  |  |  |  |  |  |  |  |  |  |  |  |
| --- | --- | --- | --- | --- | --- | --- | --- | --- | --- | --- | --- | --- | --- | --- | --- |
| *N* | 339 | 12 | 32 | 27 | 20 | 31 | 32 | 21 | 22 | 24 | 27 | 26 | 25 | 26 | 14 |
| *Na* | 27 | 3 | 7 | 4 | 6 | 6 | 4 | 5 | 5 | 5 | 7 | 17 | 9 | 7 | 3 |
| *A*_R_ | 9.627 | 3.00 | 5.46 | 2.88 | 5.53 | 5.04 | 3.80 | 4.47 | 4.33 | 4.96 | 6.45 | 11.49 | 6.89 | 5.47 | 2.97 |
| *H*_o_ | 0.434 | 0.417 | 0.406 | 0.259 | 0.600 | 0.161 | 0.281 | 0.238 | 0.500 | 0.583 | 0.704 | 0.808 | 0.560 | 0.462 | 0.000 |
| *H*_e_ | 0.806 | 0.540 | 0.695 | 0.294 | 0.724 | 0.623 | 0.514 | 0.547 | 0.613 | 0.769 | 0.787 | 0.858 | 0.779 | 0.646 | 0.265 |
| *F*_IS_ | **0.462** | 0.236 | **0.419** | 0.119 | 0.175 | **0.744** | 0.457 | **0.571** | 0.188 | 0.245 | 0.107 | 0.06 | 0.285 | 0.289 | 1 |
| EMLS04 |  |  |  |  |  |  |  |  |  |  |  |  |  |  |  |
| *N* | 351 | 13 | 31 | 30 | 22 | 32 | 30 | 25 | 24 | 25 | 27 | 27 | 25 | 26 | 14 |
| *Na* | 5 | 2 | 3 | 1 | 2 | 1 | 1 | 2 | 2 | 3 | 2 | 2 | 2 | 3 | 1 |
| *A*_R_ | 2.778 | 2.00 | 2.63 | 1.00 | 2.00 | 1.00 | 1.00 | 1.48 | 1.99 | 2.22 | 2.00 | 2.00 | 2.00 | 2.46 | 1.00 |
| *H*_o_ | 0.202 | 0.154 | 0.323 | 0.000 | 0.409 | 0.000 | 0.000 | 0.040 | 0.250 | 0.120 | 0.370 | 0.185 | 0.440 | 0.539 | 0.000 |
| *H*_e_ | 0.317 | 0.148 | 0.368 | 0.000 | 0.384 | 0.000 | 0.000 | 0.040 | 0.223 | 0.117 | 0.425 | 0.373 | 0.393 | 0.520 | 0.000 |
| *F*_IS_ | **0.361** | -0.043 | 0.125 | _ | -0.068 | _ | _ | 0 | -0.122 | -0.029 | 0.13 | 0.508 | -0.123 | -0.037 | _ |
| EMLS13 |  |  |  |  |  |  |  |  |  |  |  |  |  |  |  |
| *N* | 354 | 13 | 32 | 30 | 22 | 32 | 32 | 26 | 24 | 24 | 27 | 27 | 25 | 26 | 14 |
| *Na* | 4 | 2 | 2 | 2 | 2 | 2 | 2 | 4 | 2 | 2 | 1 | 2 | 2 | 2 | 2 |
| *A*_R_ | 2.133 | 1.92 | 2.00 | 2.00 | 1.91 | 2.00 | 2.00 | 3.43 | 2.00 | 2.00 | 1.00 | 1.84 | 2.00 | 1.72 | 2.00 |
| *H*_o_ | 0.271 | 0.077 | 0.438 | 0.367 | 0.136 | 0.375 | 0.563 | 0.308 | 0.458 | 0.333 | 0.000 | 0.037 | 0.320 | 0.000 | 0.071 |
| *H*_e_ | 0.470 | 0.077 | 0.508 | 0.463 | 0.130 | 0.437 | 0.476 | 0.434 | 0.503 | 0.422 | 0.000 | 0.107 | 0.327 | 0.075 | 0.198 |
| *F*_IS_ | **0.423** | 0 | 0.141 | 0.21 | -0.05 | 0.143 | -0.185 | 0.296 | 0.09 | 0.214 | _ | 0.658 | 0.02 | 1 | 0.649 |
| EMLS21 |  |  |  |  |  |  |  |  |  |  |  |  |  |  |  |
| *N* | 352 | 13 | 32 | 29 | 22 | 31 | 32 | 25 | 24 | 25 | 27 | 27 | 25 | 26 | 14 |
| *Na* | 3 | 1 | 1 | 1 | 2 | 1 | 1 | 1 | 2 | 1 | 1 | 1 | 1 | 1 | 1 |
| *A*_R_ | 1.101 | 1.00 | 1.00 | 1.00 | 1.80 | 1.00 | 1.00 | 1.00 | 1.50 | 1.00 | 1.00 | 1.00 | 1.00 | 1.00 | 1.00 |
| *H*_o_ | 0.003 | 0.000 | 0.000 | 0.000 | 0.000 | 0.000 | 0.000 | 0.000 | 0.042 | 0.000 | 0.000 | 0.000 | 0.000 | 0.000 | 0.000 |
| *H*_e_ | 0.009 | 0.000 | 0.000 | 0.000 | 0.089 | 0.000 | 0.000 | 0.000 | 0.042 | 0.000 | 0.000 | 0.000 | 0.000 | 0.000 | 0.000 |
| *F*_IS_ | **0.666** | _ | _ | _ | 1 | _ | _ | _ | 0 | _ | _ | _ | _ | _ | _ |

**Table S3.** *continued.*

| EMLS26 |  |  |  |  |  |  |  |  |  |  |  |  |  |  |  |
| --- | --- | --- | --- | --- | --- | --- | --- | --- | --- | --- | --- | --- | --- | --- | --- |
| *N* | 355 | 13 | 32 | 30 | 22 | 32 | 32 | 26 | 24 | 25 | 27 | 27 | 25 | 26 | 14 |
| *Na* | 8 | 5 | 5 | 6 | 5 | 2 | 4 | 4 | 4 | 4 | 4 | 3 | 3 | 3 | 2 |
| *A*_R_ | 5.934 | 4.99 | 4.50 | 5.51 | 4.47 | 2.00 | 2.90 | 3.81 | 3.95 | 3.87 | 3.39 | 2.96 | 2.74 | 1.92 | 2.00 |
| *H*_o_ | 0.468 | 0.615 | 0.656 | 0.800 | 0.546 | 0.313 | 0.188 | 0.462 | 0.542 | 0.520 | 0.482 | 0.482 | 0.600 | 0.077 | 0.286 |
| *H*_e_ | 0.787 | 0.726 | 0.682 | 0.777 | 0.650 | 0.347 | 0.231 | 0.472 | 0.713 | 0.637 | 0.405 | 0.514 | 0.548 | 0.076 | 0.254 |
| *F*_IS_ | **0.406** | 0.158 | 0.038 | -0.03 | 0.164 | 0.101 | 0.19 | 0.023 | 0.244 | 0.186 | -0.192 | 0.064 | -0.098 | -0.01 | -0.13 |
| EMLS29 |  |  |  |  |  |  |  |  |  |  |  |  |  |  |  |
| *N* | 348 | 13 | 32 | 29 | 22 | 28 | 32 | 26 | 24 | 25 | 26 | 26 | 25 | 26 | 14 |
| *Na* | 4 | 2 | 2 | 3 | 2 | 2 | 2 | 1 | 1 | 1 | 1 | 3 | 3 | 1 | 3 |
| *A*_R_ | 3.105 | 2.00 | 2.00 | 2.47 | 1.55 | 2.00 | 2.00 | 1.00 | 1.00 | 1.00 | 1.00 | 2.46 | 2.45 | 1.00 | 2.86 |
| *H*_o_ | 0.112 | 0.154 | 0.406 | 0.069 | 0.046 | 0.143 | 0.281 | 0.000 | 0.000 | 0.000 | 0.000 | 0.192 | 0.080 | 0.000 | 0.071 |
| *H*_e_ | 0.280 | 0.271 | 0.396 | 0.164 | 0.046 | 0.486 | 0.289 | 0.000 | 0.000 | 0.000 | 0.000 | 0.298 | 0.220 | 0.000 | 0.405 |
| *F*_IS_ | **0.6** | 0.442 | -0.025 | 0.584 | 0 | **0.71** | 0.028 | _ | _ | _ | _ | 0.359 | 0.64 | _ | 0.829 |
| EMLS41 |  |  |  |  |  |  |  |  |  |  |  |  |  |  |  |
| *N* | 355 | 13 | 32 | 30 | 22 | 32 | 32 | 26 | 24 | 25 | 27 | 27 | 25 | 26 | 14 |
| *Na* | 6 | 2 | 3 | 3 | 3 | 3 | 4 | 4 | 5 | 4 | 2 | 3 | 2 | 1 | 2 |
| *A*_R_ | 4.669 | 2.00 | 2.95 | 3.00 | 2.80 | 3.00 | 3.93 | 3.39 | 4.64 | 3.99 | 2.00 | 2.87 | 1.99 | 1.00 | 1.86 |
| *H*_o_ | 0.378 | 0.462 | 0.594 | 0.533 | 0.136 | 0.406 | 0.500 | 0.308 | 0.458 | 0.640 | 0.407 | 0.259 | 0.280 | 0.000 | 0.071 |
| *H*_e_ | 0.694 | 0.517 | 0.590 | 0.666 | 0.529 | 0.548 | 0.585 | 0.367 | 0.740 | 0.678 | 0.331 | 0.297 | 0.246 | 0.000 | 0.071 |
| *F*_IS_ | **0.456** | 0.111 | -0.007 | 0.202 | **0.746** | 0.261 | 0.147 | 0.163 | 0.386 | 0.058 | -0.238 | 0.129 | -0.143 | _ | 0 |
| EMLS45 |  |  |  |  |  |  |  |  |  |  |  |  |  |  |  |
| *N* | 347 | 13 | 32 | 30 | 21 | 32 | 31 | 23 | 23 | 25 | 27 | 26 | 25 | 25 | 14 |
| *Na* | 9 | 2 | 4 | 3 | 4 | 4 | 3 | 4 | 4 | 4 | 2 | 3 | 2 | 3 | 3 |
| *A*_R_ | 3.794 | 1.92 | 3.97 | 2.64 | 3.32 | 2.75 | 2.92 | 3.78 | 3.20 | 3.47 | 2.00 | 2.39 | 2.00 | 2.48 | 2.86 |
| *H*_o_ | 0.378 | 0.077 | 0.563 | 0.367 | 0.143 | 0.563 | 0.419 | 0.348 | 0.261 | 0.360 | 0.407 | 0.192 | 0.520 | 0.240 | 0.643 |
| *H*_e_ | 0.507 | 0.077 | 0.732 | 0.396 | 0.264 | 0.499 | 0.418 | 0.410 | 0.243 | 0.425 | 0.409 | 0.180 | 0.481 | 0.456 | 0.553 |
| *F*_IS_ | **0.256** | 0 | 0.234 | 0.075 | 0.464 | -0.131 | -0.003 | 0.154 | -0.078 | 0.156 | 0.003 | -0.068 | -0.083 | 0.479 | -0.17 |
|  |  |  |  |  |  |  |  |  |  |  |  |  |  |  |  |
| ***Table S3****. note: n indicates the number genotyped individuals, Na is the number of alleles, A_R_ is allelic richness (based on samples of 12 individuals), H_O_ is the observed heterozygosity, H_E_ is the unbiased expected heterozygosity, and F_IS_ is the inbreeding coefficient (significance is indicated in bold characters).* | | | | | | | | | | | | | | | |
