# Supplementary material for: Environmental versus Anthropogenic Effects on Population Adaptive Divergence in the Freshwater Snail Lymnaea stagnalis
Source: PLoS One. 2014 Sep 10;9(9):e106670. doi: 10.1371/journal.pone.0106670 (PMC4160221; doi:10.1371/journal.pone.0106670)
Supplement: Table S4 — Population genetic differentiation estimated among 14 L. stagnalis populations, on the basis of 12 SSR loci. (DOCX) [file pone.0106670.s006.docx]

**Table S4.** Population genetic differentiation estimated among 14 *L. stagnalis* populations, on the basis of 12 SSR loci.

|  | 1.OUD | 2.OOS | 3.BIE | 4.BAA | 5.CAS | 6.PUT | 7.SCH | 8.EMM | 9.KUI | 10.BUX | 11.KOE | 12.AGA | 13.HED | 14.DET |
| --- | --- | --- | --- | --- | --- | --- | --- | --- | --- | --- | --- | --- | --- | --- |
| 1.OUD |  | * | * | * | * | * | * | * | * | * | * | * | * | * |
| 2.OOS | 0.129 |  | * | * | * | * | * | * | * | * | * | * | * | * |
| 3.BIE | 0.180 | 0.124 |  | * | * | * | * | * | * | * | * | * | * | * |
| 4.BAA | 0.274 | 0.148 | 0.158 |  | * | * | * | * | * | * | * | * | * | * |
| 5.CAS | 0.405 | 0.247 | 0.291 | 0.319 |  | * | * | * | * | * | * | * | * | * |
| 6.PUT | 0.335 | 0.188 | 0.154 | 0.219 | 0.305 |  | * | * | * | * | * | * | * | * |
| 7.SCH | 0.285 | 0.202 | 0.200 | 0.302 | 0.365 | 0.146 |  | * | * | * | * | * | * | * |
| 8.EMM | 0.143 | 0.116 | 0.180 | 0.170 | 0.347 | 0.240 | 0.194 |  | NS | * | * | * | * | * |
| 9.KUI | 0.211 | 0.135 | 0.231 | 0.187 | 0.384 | 0.266 | 0.249 | 0.041 |  | * | * | * | * | * |
| 10.BUX | 0.415 | 0.283 | 0.330 | 0.300 | 0.464 | 0.352 | 0.404 | 0.335 | 0.369 |  | * | * | * | * |
| 11.KOE | 0.349 | 0.245 | 0.229 | 0.223 | 0.382 | 0.231 | 0.290 | 0.237 | 0.287 | 0.138 |  | * | * | * |
| 12.AGA | 0.293 | 0.209 | 0.231 | 0.232 | 0.416 | 0.294 | 0.306 | 0.206 | 0.262 | 0.147 | 0.081 |  | * | * |
| 13.HED | 0.475 | 0.330 | 0.418 | 0.423 | 0.548 | 0.462 | 0.455 | 0.364 | 0.412 | 0.212 | 0.247 | 0.149 |  | * |
| 14.DET | 0.348 | 0.230 | 0.290 | 0.404 | 0.478 | 0.364 | 0.440 | 0.382 | 0.395 | 0.519 | 0.449 | 0.419 | 0.597 |  |

*Lower diagonal: pairwise F_ST_ values.*

*Upper diagonal: nominal level for multiple comparisons (significance threshold adjusted to 0.000549).*
