# Supplementary material for: Environmental versus Anthropogenic Effects on Population Adaptive Divergence in the Freshwater Snail Lymnaea stagnalis
Source: PLoS One. 2014 Sep 10;9(9):e106670. doi: 10.1371/journal.pone.0106670 (PMC4160221; doi:10.1371/journal.pone.0106670)
Supplement: Table S5 — Life-history trait/parameter mean-values (SE) estimated in the laboratory-born G1 from 14 L. stagnalis populations used to test patterns of adaptive divergence (Q ST-F ST analysis). (DOCX) [file pone.0106670.s007.docx]

**Table S5**. Life-history trait/parameter mean-values (SE) estimated in the laboratory-born G_1_ from 14 *L. stagnalis* populations used to test patterns of adaptive divergence (*Q*_ST -_ *F*_ST_ analysis).

| Level of Global environmental Pressure | GEP0 | | | |  | GEP1 | | |  | GEP2 | | | | | | |
| --- | --- | --- | --- | --- | --- | --- | --- | --- | --- | --- | --- | --- | --- | --- | --- | --- |
| Pop (Habitat / Genetic cluster) | 9.KUI (D/W) | 1.OUD (P/W) | 5.CAS (P/W) | 13.HED (P/E) |  | 14.DET (P/E) | 7.SCH (C/W) | 10.BUX (C/E) |  | 2.OOS (C/W) | 3.BIE (C/W) | 11.KOE (C/E) | 12.AGA (D/E) | 4.BAA (D/W) | 6.PUT (D/W) | 8.EMM (D/W) |
| *Growth* |  |  |  |  |  |  |  |  |  |  |  |  |  |  |  |  |
| Hatching size (mm) | 2.91 | 3.06 | 3.11 | 2.99 |  | 3.33 | 3 | 2.93 |  | 3.06 | 3 | 2.93 | 2.91 | 2.85 | 3.05 | 2.96 |
|  | (0.04) | (0.06) | (0.04) | (0.03) |  | (0.05) | (0.04) | (0.03) |  | (0.04) | (0.04) | (0.04) | (0.03) | (0.06) | (0.04) | (0.04) |
| Parameter *b* | 1.5 | 1.4 | 1.4 | 1.5 |  | 1.4 | 1.5 | 1.4 |  | 1.4 | 1.4 | 1.5 | 1.5 | 1.6 | 1.4 | 1.5 |
|  | (0.02) | (0.03) | (0.02) | (0.02) |  | (0.03) | (0.02) | (0.02) |  | (0.02) | (0.02) | (0.02) | (0.03) | (0.05) | (0.03) | (0.04) |
| Parameter *k* | 0.986 | 0.985 | 0.986 | 0.987 |  | 0.987 | 0.986 | 0.985 |  | 0.986 | 0.986 | 0.986 | 0.986 | 0.985 | 0.986 | 0.987 |
|  | (2.2e-4) | (5.6e-4) | (2.2e-4) | (1.8e-4) |  | (4.4e-4) | (2.7e-4) | (2.6e-4) |  | (1.6e-4) | (2.3e-4) | (2.2e-4) | (2.9e-4) | (8.0e-4) | (2.7e-4) | (2.7e-4) |
| Parameter *A* (mm) | 31.1 | 31.4 | 33.2 | 33.8 |  | 39.5 | 31.1 | 30.2 |  | 31.6 | 31.7 | 30.7 | 30.5 | 27.8 | 30.4 | 32.9 |
|  | (0.6) | (0.4) | (0.5) | (0.4) |  | (0.8) | (0.4) | (0.4) |  | (0.5) | (0.5) | (0.5) | (0.4) | (1.1) | (0.5) | (0.6) |
| Size at 119 days (mm) | 18 | 18.8 | 18.5 | 17.9 |  | 18.8 | 17.5 | 18.2 |  | 18.3 | 18.2 | 17.4 | 17.8 | 17 | 17.9 | 18.4 |
|  | (0.1) | (0.2) | (0.2) | (0.1) |  | (0.2) | (0.1) | (0.1) |  | (0.1) | (0.1) | (0.1) | (0.1) | (0.3) | (0.1) | (0.1) |
| *Reproduction* |  |  |  |  |  |  |  |  |  |  |  |  |  |  |  |  |
| Ability to lay eggs (%) | 100 | 100 | 58.3 | 94.4 |  | 69.4 | 100 | 97.2 |  | 97.2 | 100 | 97.2 | 97.2 | 91.7 | 97.2 | 100 |
| Time to oviposition (days) | 3.8 | 2.7 | 15.6 | 5.1 |  | 12.3 | 5.5 | 3.9 |  | 5.1 | 5.4 | 4.3 | 3.2 | 6.8 | 4.6 | 4.4 |
|  | (0.4) | (0.3) | (1.8) | (0.9) |  | (1.9) | (0.4) | (0.8) |  | (0.8) | (0.5) | (0.8) | (0.5) | (1.6) | (0.8) | (0.4) |
| Number of clutches /snail | 3 | 5.8 | 1.2 | 2.9 |  | 2 | 2.1 | 3.8 |  | 3.3 | 2.8 | 3.4 | 3.7 | 4.1 | 2.9 | 2.9 |
|  | (0.2) | (0.2) | (0.2) | (0.3) |  | (0.4) | (0.2) | (0.3) |  | (0.3) | (0.2) | (0.2) | (0.2) | (0.5) | (0.2) | (0.2) |
| Number of eggs / snail | 101.8 | 149.5 | 51.4 | 107 |  | 55.7 | 99.3 | 144.1 |  | 145.1 | 143.2 | 118 | 110.6 | 105.2 | 111.1 | 112.1 |
|  | (8.2) | (5.9) | (9.6) | (9.3) |  | (11.2) | (9.4) | (11.4) |  | (14.6) | (8.5) | (7.3) | (7) | (12.9) | (7.6) | (8.3) |
| Clutche size (eggs) | 34.6 | 26.2 | 45.4 | 36.5 |  | 27.7 | 47.1 | 38 |  | 44.3 | 50.8 | 36.2 | 30.6 | 26.1 | 37.4 | 39.5 |
|  | (1.4) | (0.9) | (2.8) | (1.6) |  | (1.9) | (2.7) | (1.1) |  | (1.8) | (2.1) | (1.3) | (1.2) | (1.6) | (1.8) | (1.7) |
| Hatching rate (%) | 97.5 | 97.5 | 94 | 92.6 |  | 85.1 | 95.4 | 96.4 |  | 97.1 | 92.4 | 97.4 | 95 | 83 | 96.7 | 96 |
|  | (0.7) | (0.6) | (2.2) | (1.6) |  | (2.4) | (1.4) | (1.1) |  | (0.5) | (1.5) | (0.5) | (1) | (2.7) | (0.6) | (0.5) |

*Habitat: P = Pond, D = Ditch, C = Channel; Genetic cluster: W = West, E = East. A is asymptotic size, b is related to size at t_0_, k is related to*

*growth rate.*
